# Investigating the interplay between segregation and integration in developing cortical assemblies

**DOI:** 10.3389/fncel.2024.1429329

**Published:** 2024-09-12

**Authors:** Valerio Barabino, Ilaria Donati della Lunga, Francesca Callegari, Letizia Cerutti, Fabio Poggio, Mariateresa Tedesco, Paolo Massobrio, Martina Brofiga

**Affiliations:** ^1^Department of Informatics, Bioengineering, Robotics and Systems Engineering (DIBRIS), University of Genova, Genova, Italy; ^2^Neurofacility, Istituto Italiano di Tecnologia, Genova, Italy; ^3^National Institute for Nuclear Physics (INFN), Genova, Italy; ^4^ScreenNeuroPharm S.r.l, Sanremo, Italy

**Keywords:** cortical networks, segregation, integration, micro-electrode arrays, PDMS device, connectivity

## Abstract

**Introduction:**

The human brain is an intricate structure composed of interconnected modular networks, whose organization is known to balance the principles of segregation and integration, enabling rapid information exchange and the generation of coherent brain states. Segregation involves the specialization of brain regions for specific tasks, while integration facilitates communication among these regions, allowing for efficient information flow. Several factors influence this balance, including maturation, aging, and the insurgence of neurological disorders like epilepsy, stroke, or cancer. To gain insights into information processing and connectivity recovery, we devised a controllable *in vitro* model to mimic and investigate the effects of different segregation and integration ratios over time.

**Methods:**

We designed a cross-shaped polymeric mask to initially establish four independent sub-populations of cortical neurons and analyzed how the timing of its removal affected network development. We evaluated the morphological and functional features of the networks from 11 to 18 days *in vitro* (DIVs) with immunofluorescence techniques and micro-electrode arrays (MEAs).

**Results:**

The removal of the mask at different developmental stages of the network lead to strong variations in the degree of intercommunication among the four assemblies (altering the segregation/integration balance), impacting firing and bursting parameters. Early removal (after 5 DIVs) resulted in networks with a level of integration similar to homogeneous controls (without physical constraints). In contrast, late removal (after 15 DIVs) hindered the formation of strong inter-compartment connectivity, leading to more clustered and segregated assemblies.

**Discussion:**

A critical balance between segregation and integration was observed when the mask was removed at DIV 10, allowing for the formation of a strong connectivity among the still-separated compartments, thus demonstrating the existence of a time window in network development in which it is possible to achieve a balance between segregation and integration.

## Introduction

The human brain, with its intricate network of neurons and synapses, stands as a marvel of biological complexity. Within this extraordinary organ, two fundamental principles, segregation and integration, orchestrate the symphony of cognitive processes, behaviors, and perceptions that define human experience (Cohen and D’Esposito, 2016; Lord et al., 2017). As confirmed by studies reporting structural analyses of brain networks carried out on datasets describing the cerebral cortex of mammalian animal models (e.g., rat, cat, monkey), brain areas were found to be neither completely connected with each other nor randomly linked (Sporns and Bullmore, 2014). Instead, their interconnections showed a specific and intricate organization ruled by a delicate balance between segregation and integration.

Segregation entails the specialization of distinct brain regions for specific functions, allowing for efficient processing of information. For example, the occipital lobe is dedicated to visual processing (Rehman and Al Khalili, 2023), while the prefrontal cortex governs executive functions such as decision-making and planning (Stuss and Benson, 1984). Segregation enables the brain to carry out diverse cognitive processes in parallel, increasing efficiency and allowing for complex computations to unfold seamlessly. Disruptions in segregation can lead to profound cognitive impairments, as observed in neurological conditions such as stroke or traumatic brain injury (Gratton et al., 2012; Guo et al., 2019; Wang et al., 2020) where damage results in reduced connectivity and clusterization within specific brain regions, causing deficits in sensory perception, motor function, and language processing.

Integration facilitates the exchange of information between segregated regions, enabling the brain to generate coherent perceptions and adaptive responses to the environment (Lord et al., 2017). At its essence, integration embodies the interplay between segregated brain regions, fostering the combination of sensory inputs, memories, emotions, and cognitive functions into unified experiences. This intricate process transcends mere summation of neural activity; rather, it involves the complex orchestration of neuronal firing patterns (Shine et al., 2019; Luppi et al., 2023), neurotransmitter release (Coronel-Oliveros et al., 2021), and synaptic plasticity across distributed networks (Bassi et al., 2019). Understanding the mechanisms and consequences of integration can help elucidate the neuronal basis of different phenomena, from consciousness and self-awareness to psychopathology and neurodegeneration. Dysfunctions in integration are implicated in autism spectrum disorder (Just et al., 2012) and in many psychiatric conditions, including schizophrenia (Stephan et al., 2009), and depression (Northoff et al., 2011), further underscoring the importance of unraveling its principles for developing targeted therapeutic approaches.

In physiological conditions, the balanced cooperation of segregation and integration optimizes brain functions and maximizes information transmission capabilities. In this context, *in vitro* neuronal models offer a powerful tool to design simplified yet physiologically relevant systems that account for key aspects of brain development and function. They allow for precise manipulation and observation of neuronal circuits, providing insights into how segregation and integration processes evolve over time and in response to various stimuli.

Over the years, various approaches and techniques have been developed and tested to create engineered *in vitro* neuronal cultures that replicate the segregation and integration properties of the *in vivo* microenvironment. Many studies have focused on manipulating the chemical and physical properties of the substrate to generate interconnected modules (Marconi et al., 2012; Shein-Idelson et al., 2016; Yamamoto et al., 2018). A common technique is micro-contact printing, which creates areas that either repel or promote cell adhesion. However, it requires extensive time and effort (Kaehr et al., 2004; Yamamoto et al., 2011), and the integrity of the network structure depends on the desorption and adsorption dynamics of surface molecules. This represents an intrinsic limit since it does not allow the definition of medium-size cellular populations. A valuable option is the use of poly-dimethyl-siloxane (PDMS) devices. These systems are able to segregate cell cultures in specific areas and to facilitate the interconnection of different modules through the presence of microchannels (Virlogeux et al., 2018; Brofiga et al., 2021; Chen et al., 2023; Wang et al., 2024). However, this organization is permanent and does not allow for the modulation of the levels of integration and segregation over time and the consequent correlation with the emerging dynamical states of the network. The capability to regulate the levels of integration over time is crucial not only to better understand the evolution of the network but also to investigate and emulate the possible onset and progression of pathologies that disrupt such equilibrium.

In this perspective, we designed a cross-shaped polymer device able to dynamically segregate four distinct neuronal populations: by removing this structure at different time points over development, we were able to guide the structure of the network and promote either segregation, integration, or their balance. We employed immunofluorescence techniques to determine structural neuronal connectivity and micro-electrode arrays (MEAs) recordings to infer the different dynamics expressed as a function of the level of segregation and integration. We demonstrated that removing the physical constraint at different stages of development resulted in significant changes in how the assemblies communicated with each other. We found that a good balance between segregation and integration was achieved when the masks were removed at DIV 10 (RD10), allowing for the formation of strong connections while still maintaining some degree of separation among assemblies. When the cross-shaped masks were removed early on (DIV 5, RD05), the networks behaved similarly to homogeneous networks (controls). If the physical constraint were removed at late stages of development (DIV 15, RD15), the sub-populations struggled to establish a strong inter-compartment connectivity, both morphological and functional, leading to more segregated sub-populations. In other words, our research suggests that there exists a crucial time frame during the developmental phase of an *in vitro* neuronal network, where it is feasible to strike an equilibrium between distinct assemblies while reinforcing the connections between them.

## Materials and methods

### Cell culture preparation

Dissociated cortical cultures were obtained from Sprague–Dawley embryonic rats at gestational day 18–19 (E18–E19), in compliance with European Animal Care Legislation (2010/63/EU), the Ministry of Health’s legislative decree (D.L. 116/192), and the University of Genova guidelines (Prot. 75F11.N.6JI, 08/08/18). Cortical tissue was extracted and dissociated following the procedure described in Brofiga et al. (2023). First, it underwent an enzymatic treatment through a solution comprising 0.125% Trypsin and 0.05% DNAse, diluted in Hank’s Balanced Salt Solution (HBSS, Sigma Aldrich, W/O calcium and magnesium), for approximately 18–20 min in a water bath at 37°C. Digestion was halted by adding Neurobasal medium (Gibco Invitrogen) supplemented with 10% fetal bovine serum (FBS, Sigma-Aldrich). Tissue was then further dissociated through mechanical trituration using a fine-tipped Pasteur pipette. The resulting cellular suspension was diluted in Neurobasal medium, enriched with 2% B-27 Supplement (Gibco Invitrogen), 1% stable L-Glutamine (GlutaMAX 100x, Gibco Invitrogen), and 1% Penicillin-Streptomycin Solution (PenStrep, Gibco Invitrogen). No antimitotic drug was introduced to avoid glial proliferation, as glial cells play a crucial role in the healthy development of neuronal populations (Pfrieger, 2010). Cells were plated at the final density of 1′500 cells/mm^2^ directly onto the surfaces of 120-channel micro electrode arrays (MEAs, Multi Channel Systems, Germany, MCS), presterilized and precoated with poly-L-ornithine (100 μg/mL, Sigma Aldrich). The biological samples were then placed in incubator at 37°C, 5% CO_2_, and 95% humidity. Five days post-plating, half of the Neurobasal medium was replaced with BrainPhys medium (STEMCELL Technologies), supplemented with 2% NeuroCult SM1 (STEMCELL Technologies), 1% GlutaMAX, and 1% PenStrep solution. The medium was changed twice a week to facilitate the organization of neurons into a morphologically and functionally mature network within three weeks.

### Polymeric device

**Figure 1:**
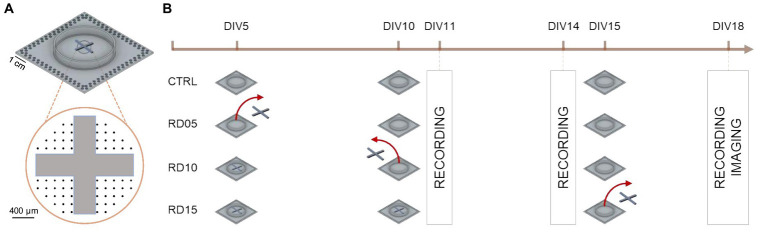
Schematics of the experimental design and data collection timeline. **(A)** Positioning of the cross-shaped PDMS mask on the MEA. The black dots indicate the electrode layout. **(B)** Timeline that summarizes the experimental steps, highlighting the removal day of the cross-shaped mask in each configuration (with a red arrow) and the days when data (MEA recordings, images) were collected (boxes).

Cross-shaped poly-dimethyl-siloxane (PDMS) masks were used to engineer the network connectivity and regulate the balance between integration and segregation. The cross-shaped masks were realized as in Brofiga et al. (2023). They consisted of two thin rectangular-shape barriers of equal dimensions measuring 2 mm in length, 0.6 mm in width, and 0.3 mm in thickness (Figure 1A). They were fabricated from a PDMS prepolymer and curing agent mixture (Sylgard 184, Sigma Aldrich) at a 10:1 (w/w) ratio and underwent polymerization at 80°C in a dry oven. Following sterilization in 70% ethanol overnight, the cross-shaped masks were aligned and plated onto pre-sterilized and coated planar MEAs in a reversible manner. These devices allowed the splitting of the active area of the MEA into four sub-regions, thereby establishing four neuronal compartments (4C). No morphological connections arose among the neurons plated in the four compartments until the mask’s removal.

### Electrophysiology

The spontaneous electrophysiological activity of the neuronal networks was recorded using the MEA2100 system (MCS) at a sampling frequency of 10 kHz. Recordings were conducted with the MEAs placed upon the heated (37°C) amplifier location. This positioning allowed the cultures to recover from the thermal and mechanical stress induced by the transfer from the incubator, with recordings starting approximately 15 min after placement. To maintain stable conditions and prevent evaporation and changes in the pH of the medium, a constant slow flow of humidified gas (5% CO_2_, 20% O_2_, 75% N_2_) was maintained over the MEA. Data recording was carried out using the MC_Rack software (MCS) after 11, 14, and 18 days *in vitro* (DIVs). Offline data analysis was performed using MATLAB (The Mathworks, Natick, United States of America) and Python (Van Rossum and Drake, 1995).

### Immunofluorescence staining

Immunofluorescence staining was carried out at DIV 18 for cortical cultures in which the cross-shaped mask was removed after 5, 10, and 15 days *in vitro*. Cell cultures were fixed with 4% paraformaldehyde (PFA, Sigma-Aldrich) at pH 7.4 for 10 min at room temperature. Permeabilization was achieved by treating the cells with phosphate buffer solution (PBS, Sigma-Aldrich) containing 0.1% Triton-X100 (Sigma-Aldrich) for 10 min at room temperature. To minimize non-specific antibody binding, cells were left for 40 min in a blocking buffer solution (BBS) consisting of a PBS solution with the addition of 5% fetal bovine serum (FBS, Sigma Aldrich). Subsequently, cells were incubated with primary antibodies diluted in BBS for 2 h at room temperature. The primary antibodies used were Tau (axon microtubule-associated protein, mouse monoclonal 1:500, Synaptic System) and MAP2 (dendritic microtubule-associated protein, rabbit polyclonal 1:500, Synaptic System). Cultures were then washed three times with PBS and exposed for 40 min at room temperature to secondary antibodies: Alexa Fluor 488 (1:700, Invitrogen) and Alexa Fluor 546 (1:1000, Invitrogen), Goat anti-mouse or Goat anti-rabbit. Fluorescent images were captured with Leica TCS SP5 AOBS Tandem DM6000 upright microscope coupled with objective Leica HCX IRAPO L 25× 0.95 N.A., water (Leica Microsystems S.r.l. Italy), and analyzed with ImageJ.

### Dataset

Spontaneous electrophysiological activity was recorded at three time points, namely DIV 11, DIV 14, and DIV 18. Each recording (lasting 20 min) involved cultures in which the cross-shaped mask was removed at DIV 5 (RD05), DIV 10 (RD10) and DIV 15 (RD15). Relative controls comprised homogeneous cortical cultures in which the cross-shaped mask was not applied to the MEAs. The *n* = 117 recordings came from *n* = 6 preparations. Figure 1B depicts a sketch of the experimental pipeline and Table 1 summarizes the entire dataset, pointing out the kind of experiment, the DIV of recording, and the day of cross-shaped mask removal.

**Table 1:** Dataset expressed as number of MEAs for each configuration and DIV.

	Control	RD05	RD10	RD15
DIV 11	11	8	8	6
DIV 14	11	13	12	14
DIV 18	8	10	6	10

### Data analysis

#### Spike and burst analysis

Spike occurrences were identified using the Precision Time Spike Detection (PTSD) algorithm (Maccione et al., 2009). This method requires the definition of three parameters: (I) a differential threshold for each electrode, computed as eight times the standard deviation of the signal’s biological and thermal noise; (II) the peak lifetime period set at 2 ms; (III) the refractory period set at 1 ms. Data were not subjected to spike sorting due to the nature of bursting events. During such events, a global increase in activity leads to a rapid succession of spikes with varied and overlapping shapes, making sorting unreliable (Wagenaar et al., 2006). Once spikes were detected, burst detection was conducted using the string method introduced in Chiappalone et al. (2005). This algorithm consists of (i) establishing the minimum number of spikes within a string to define a burst and (ii) determining the maximum interspike interval that distinguishes consecutive spikes as part of a burst. These values were set at 5 and 100 ms, respectively. Macroscopic spiking and bursting behaviors of the networks were evaluated in terms of the mean number of spikes per second (mean firing rate, MFR), the mean number of bursts per minute (mean bursting rate, MBR), the spike count over the duration of bursts (mean frequency intra burst, MFIB), and the percentage of spikes occurring outside bursts (random spikes, RS). An electrode was considered active if its MFR value was higher than 0.1 spikes/s. During the evaluation process, each compartment in every MEA was assessed separately. In the case of the controls, four “virtual” compartments were considered independently. Each compartment is made up of about 15 electrodes.

#### Network burst analysis

The overall bursting activity of the network was extracted by adapting the algorithm in Van Pelt et al. (2004a, 2004b). First, the network activity was binned into 25 ms intervals. Next, the number of active sites and spikes within those sites were computed, and finally, their product was evaluated: if it was higher than 5% of the maximum recorded value, a network burst was identified. Additionally, a minimum inter-network burst interval of 80 ms was established to ensure a clear distinction between network bursts. From the network burst detection, we extracted the network burst duration (NBD, i.e., the temporal extension of these events), and we defined the initiation site using the following workflow: for each network burst, we identified an active compartment if at least 3 electrodes were involved in the event (20% of the electrodes of the cluster). Then, the temporal instant and location of the electrode from which the network activity began were identified and stored. Finally, considering the minimum delay of each “follower” electrode, we evaluated the compartment activation.

#### Functional connectivity analysis

The statistical relationship between couples of neurons was extracted from their functional connectivity by adapting the total spiking probability edges algorithm (De Blasi et al., 2019). After computing the connectivity matrix (CM), all the values that did not accurately reflect functional connections were discarded. This was achieved by applying a spatial filter to preserve only the functional connections with propagation speeds falling within the range of 30 to 300 mm/s (Jacobi and Moses, 2007) and by introducing an independent hard threshold defined as:

(1) $${th}_{CM}=\mu \pm \sigma$$

where *μ* and *σ* are the mean and the standard deviation of all non-zero elements in CM. From the thresholded connectivity matrix (TCM), we evaluated the node degree by summing the connections formed by each electrode, accounting for both incoming and outgoing ones. The functional connections were labeled as either “intra” or “inter” based on whether they occurred within or among compartments, and the ratio between them was extracted. This analysis was repeated for the evaluation of the “strong” connections, identified by reapplying the threshold (Equation 1) with the same values of *μ* and *σ* to TCM. Finally, we quantified the network segregation as a function of the clustering coefficient value of each node (Equation 2, CC). This was achieved by adapting the definition in Boschi et al. (2021) as follows:

(2) $${CC}_{i}=\frac{\sum_{j}1/d_{ij}^{2}}{\frac{k_{i}\left(k_{i}-1\right)}{2}}$$

where *d_ij_* is the distance (in mm) between node *i* and *j* and *k_i_* is the number of connections of the *i*th node.

For the comparison of probability distribution of the inter and intra length of the connections, namely A and B defined in the same domain Ҡ, we used the Bhattacharyya distance (Equation 3, Kailath, 1967):

(3) $$BC=-\log\left(\underset{x\in \kappa }{\sum }\sqrt{A\left(x\right)\cdot B\left(x\right)}\right)$$

### Statistical analysis

Statistical analysis was performed using non-parametric Kruskal–Wallis test, since data did not follow a normal distribution (evaluated by the Kolmogorov–Smirnov normality test). Differences were considered statistically significant when *p* < 0.05.

## Results

We explored the role of different segregation and integration levels of four cortical sub-populations exploiting micro-electrodes arrays (MEAs). Specifically, we characterized the electrophysiological properties of the 4 assemblies when interconnected at different time points, namely 5 (RD05), 10 (RD10) and 15 (RD15) days after removal of the cross-shaped mask. Moreover, we performed the same analysis on the controls (CTRL) where no physical constraint was imposed.

### Evaluation of the structural segregation and integration levels

**Figure 2:**
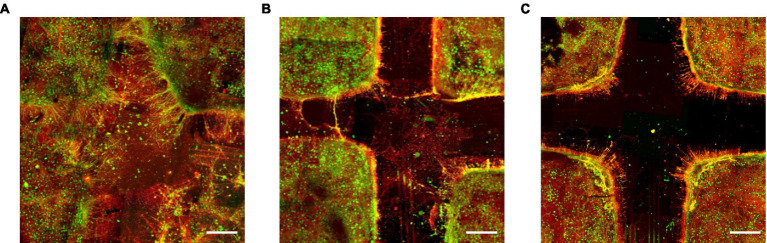
Effect of the timing of the mask removal on the inter-compartment structural connections. **(A)** RD05, **(B)** RD10, **(C)** RD15 configurations at DIV 18. Green represents neuronal soma and dendrites (anti-MAP2) and red axons and dendrites (anti-Tau): where the two markers overlap the dendrites looks yellow-orange. Scale bar: 200 μm.

Immunofluorescence techniques are a valid tool for inferring structural information about neuronal connectivity. To visualize inter-compartment links, we used anti-MAP2 (green) to label dendrites and neuronal soma and anti-Tau (red) to label dendrites and axons. At DIV 18, RD05, RD10, and RD15 were fixed and labeled to evaluate whether each configuration could effectively interconnect its segregated compartments. Figure 2A shows clearly that RD05 was able to fully interconnect the segregated compartments. After removing the cross-shaped mask, the four sub-populations filled the area among them completely, as evidenced by the extensive development of neurite arborization (both dendritic and axonal). Additionally, cell migration occurred as cell bodies did not remain confined to their original compartment but spread out to occupy the uncovered area. In this sense, RD05 lost the property of segregation. Notably, RD10 did not achieve the same level of neuritic arborization and cell migration as RD05, but axons were still visible among compartments, suggesting a good trade-off between segregation and integration among the neuronal clusters (Figure 2B). In contrast, in RD15 networks, no connections formed among compartments (Figure 2C), suggesting a lack of integration. To visualize inter-compartment links, we used anti-MAP2 (green) to label dendrites and neuronal soma and anti-Tau (red) to label dendrites and axons. At DIV 18, RD05, RD10, and RD15 were fixed and labeled to evaluate whether each configuration could effectively interconnect its segregated compartments. Figure 2A shows clearly that RD05 was able to fully interconnect the segregated compartments. After removing the cross-shaped mask, the four sub-populations filled the area among them completely, as evidenced by the extensive development of neurite arborization (both dendritic and axonal). Additionally, cell migration occurred as cell bodies did not remain confined to their original compartment but spread out to occupy the uncovered area. In this sense, RD05 lost the property of segregation. Notably, RD10 did not achieve the same level of neuritic arborization and cell migration as RD05, but axons were still visible among compartments, suggesting a good trade-off between segregation and integration among the neuronal clusters (Figure 2B). In contrast, in RD15 networks, no connections formed among compartments (Figure 2C), suggesting a lack of integration.

### Different segregation and integration levels play a key role in network maturation in terms of firing and bursting features

In the previous section, it was evident how removing the cross-shaped mask affected communication among the populations, as highlighted by the differences in physical connections among them (Figure 2). The configurations (CTRL, RD05, RD10, and RD15) differed in several aspects, including the number of cells forming the overall network and the quantity and strength of inputs each assembly received over time. Therefore, we analyzed the networks in terms of spiking and bursting parameters as a function of their development to determine whether the different configurations could achieve and sustain similar levels of dynamic richness.

**Figure 3:**
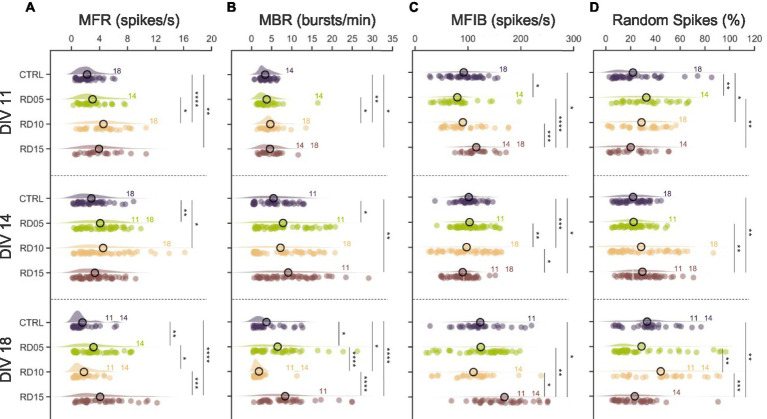
Spiking and bursting parameters. **(A)** Mean firing rate (MFR), **(B)** mean bursting rate (MBR), **(C)** mean frequency intra burst (MFIB), and **(D)** percentage of random spiking of the four different configurations (control, cross removal day at DIV 5, 10, and 15) over development (from DIV 11 to DIV 18). The statistical differences over development within the same configuration are indicated with the following abbreviation: 11, 14, and 18 if there was a statistical difference with DIV 11, DIV 14, and DIV 18, respectively. Refers to 0.01 < *p* < 0.05, * to 0.001 < *p* < 0.01, *** to 0.0001 < *p* < 0.001, and **** to *p* < 0.0001 Kruskal–Wallis non-parametric test. The *p*-values are reported in Supplementary Figures S3A,B and Supplementary Figures S4A,B.

At DIV 11, controls and RD05 networks displayed similar dynamics in terms of spiking (Figure 3A) and bursting (Figure 3B) rates; however, the distribution of the spikes inside (Figure 3C, mean frequency intra burst) and outside (Figure 3D, percentage of random spiking) the burst was slightly different. Additionally, the data exhibited greater variability in RD05 than controls, which could be attributed to the larger number of cells in the control group. Notably, RD10 emerged as the configuration showing the highest rates at this development stage (DIV 11): the removal of the physical constraints the day before the recordings brought the establishment of new disorganized inputs to the assemblies, thereby increasing the level of network activity (Okujeni et al., 2017; Joo et al., 2018; Ludl and Soriano, 2020; Tibau et al., 2020). Instead, in RD15, the network was still composed of four independent assemblies of fewer cells, resulting in four more mature networks characterized by high MFR and MBR and packed bursts (in terms of MFIB and Random Spikes).

At DIV 14, the increasing trend of the MFR, MBR, and MFIB of the controls and RD05 networks (Figures 3A–C, second row, Supplementary Figures S1, S2) suggested ongoing development and maturation of the populations. Additionally, the trend of the RD10 configuration also indicated network maturation, albeit less pronounced. This was shown by an increase in MBR and MFIB values, although it was not statistically significant (Supplementary Figures S1, S2). The difference was statistical (Supplementary Figure S3) in the case of controls (MBR) and RD05 (all parameters, with respect to DIV 11). The more marked effect in RD05 could be a result of the different stages of development (9 days after constraint removal in RD05 versus a 14 day network growth in the controls). The activity was rapidly organizing into bursts, as reflected by the decreasing percentage of random spiking (Figure 3D). In contrast, the RD15 configuration differed from the previous ones. The physical constraint was still present at DIV 14, and the four assemblies were still independent (i.e., not morphologically connected) and relatively small with respect to the other configurations. As a result, by DIV 11 the networks had already reached a higher maturation level relative to the controls (Figure 3B). By DIV 14, this increased level of maturation was still noticeable, as evidenced by the MBR, which was statistically higher compared to controls (Figure 3B, second row).

We observed a similar behavior in the controls at DIV 18, when the networks’ spiking and bursting rates started to decrease. The RD05 networks also exhibited a similar pattern, although with a less marked trend and more variability in the data (Supplementary Figures S1, S2). This suggested that the metrics of RD05 networks mirrored the progression of the controls but with a time delay, in accordance with the 5 day gap that elapsed between the formation of a fully connected network. At DIV 18, RD10 stood out among the considered configurations, displaying moderate intra-population connectivity yet allowing sufficient time for robust inter-compartment connections to develop (Figure 2B), thus achieving a balance between network integration and segregation. This balance, combined with the network maturation process, led to changes in firing and bursting rates. The high level of maturation resulted in a decrease both in MFR and MBR (Chiappalone et al., 2006) as well as in an increase in MFIB compared to DIV 14 (Wagenaar et al., 2006) (Supplementary Figure S1). This is likely due to greater integration within the network compared to previous DIVs. Additionally, there was evidence of segregation in the form of a more scattered percentage of random spikes, both compared to DIV 14 (Supplementary Figure S1) and to other configurations (Figure 3D). This effect was not observed in the case of RD15. DIV 18 identified the initial stage where the four assemblies were connected, and as a result, they were still in a disrupted environment (since the physical constraint was removed only 3 days prior and the assemblies had already reached a high level of maturation). The newly established inputs could not create robust connectivity among compartments, and consequently, the overall network frequency remained unchanged compared to previous days (Supplementary Figure S1), with only the internal distribution of the spikes being affected, resulting in a more compact burst (Figure 3C).

### Different segregation and integration levels generate different activation patterns in population events

**Figure 4:**
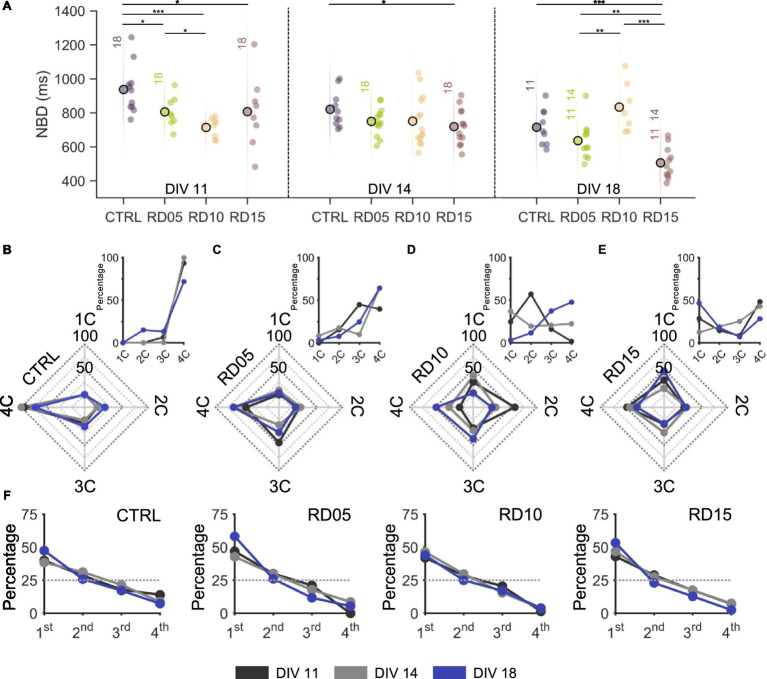
Network burst activity propagation as a function of the mask removal day. **(A)** Network burst duration of the four different configurations (control, removal day at DIV 5, 10 and 15) over development (from DIV 11 on the left to DIV 18 on the right). The statistical differences over development within the same configuration are indicated with the following abbreviation: 11, 14, and 18 if there was a statistical difference with DIV 11, DIV 14, and DIV 18, respectively. * refers to 0.01 < *p* < 0.05, ** to 0.001 < *p* < 0.01, and *** to *p* < 0.001 Kruskal–Wallis non-parametric test. **(B–E)** Radar plots showing the percentage of times network bursts involve from one single (1C) to all four (4C) compartments over development (from DIV 11 to DIV 18) in **(B)** controls, **(C)** RD05, **(D)** RD10, and **(E)** RD15 configurations. The insets offer a different visualization of the same quantity for the sake of clarity. **(F)** Percentage of times a compartment is the initiator of a network burst event over development (from DIV 11 to DIV 18) in controls, RD05, RD10, and RD15 configurations. We counted the times that the leader (initiating electrode) of a network event fell within the different compartments and consequently ordered them in a hierarchical way so that the compartment that initiated the most network events was labelled as 1st, up to the last which was labelled as 4th. The dotted grey line indicates the random condition where there is not a leader compartment, i.e., all compartments have the same probability of giving rise to a network burst. The different development conditions are color-coded as in the legend. The *p*-values are reported in Supplementary Figure S3C and Supplementary Figure S4C. Supplementary Figure S5 reports the same results expressed as a function of development.

The collective activity of the overall network and its propagation were inevitably influenced by the different levels of integration among compartments driven by our protocol throughout development. Firstly, we evaluated the durations of the network bursts (NBD, Figure 4A) as an indicator of the degree of development of the networks: population events became shorter and with prompt onsets during their maturation (Van Pelt et al., 2004a, 2004b). In agreement with this consideration, controls, RD05, and RD15 configurations showed a descending trend over the recording days. It is worth noticing that RD05 had statistically lower values than the controls at DIV 11 (Supplementary Figure S4), a difference that decreased already at DIV 14 and was maintained in the following day, confirming the observation of the previous paragraph that the maturation of RD05 networks closely followed the trend of the controls, only delayed of a few days. The behavior of RD15 was similar. At DIV 11, the four separated smaller homogeneous networks had shorter NB than the controls, suggesting that fewer-cell populations had a faster maturation. Moreover, RD15 produced the shortest NB at DIV 18, which might indicate that the network was not able to create sufficient inter-compartment connections. As in the case of spiking and bursting parameters, RD10 stood out showing an opposite behavior with respect to the other configurations (Supplementary Figure S2). Its NBD values were the lowest at DIV 11 (also among the configurations), with a very compact distribution around the mean. Over development, the average NBD increased, and the compartments displayed more variability (Supplementary Figure S2).

Furthermore, we analyzed the number of sub-populations involved in each network burst, from one (1C) to all (4C) compartments (Figures 4B–E). Independently of the day of recording, nearly all NBs of controls (Figure 4B) involved the entire network, suggesting that a full-grown connectivity among all compartments had been established at early maturation stages and was maintained over development. RD05 networks (Figure 4C) produced network bursts that involved equally 3C and 4C at DIV 11, indicating ongoing development of inter-compartment connections. At DIV 14 and DIV 18, the peak percentage shift towards 4C was a clear indication that a fully interconnected network had formed with an increased level of integration, comparable to the one of controls. This trend of population activity spreading onto more compartments over time was even clearer for RD10 (Figure 4D): at DIV 11, network events involved mostly two compartments; at DIV 14, NBs were more varied in the number of involved compartments, resulting in a uniform distribution; at DIV 18, the peak percentage moved towards 3C and 4C, similarly to RD05, but with lower percentages suggesting that segregation was maintained to some extent. Concerning the number of involved compartments, RD15 differed from the other configurations (Figure 4E). In general, the percentages were lower (never over 50%), indicating that there was not a predominant mode of propagation of the population activity. When the physical constraint was still in place (DIV 11 and DIV 14), this was justified by the fact that the coactivation of more compartments was mostly random. After the removal of the cross, the network was unable to effectively connect all compartments, which remained segregated, and therefore no predominant mode was found in the number of involved compartments. This consideration was also confirmed by the fact that NBs involved mostly one compartment at DIV 18.

Another metric considered was the presence of a sub-population leader, defined as the first compartment to fire during a network burst (Figure 4F). Across configurations and over time, there were no statistical differences. However, a leader always emerged among compartments, in accordance with previous evidence: a marked difference was always present with respect to the expected curve (random uniform distribution at 25%) (Brofiga et al., 2023).

### RD10 establishes a good trade-off between segregation and integration

The physical constraint and its removal at different development stages drove the connectivity, leading the assemblies to form either a uniform network or separated modules that communicate with each other at different levels of efficacy (i.e., coupling strength). In the previous paragraphs, we examined the impact of this physical modulation of the networks both in terms of spiking and bursting dynamics (Figure 3) and of the networks’ ability to recreate active circuits that transmit information involving all assemblies (Figure 4). We also demonstrated that these differences were supported by different dendritic and axonic growth over time in the different configurations (Figure 2). To formally support these observations, we inferred the functional connectivity analysis of the different configurations.

**Figure 5:**
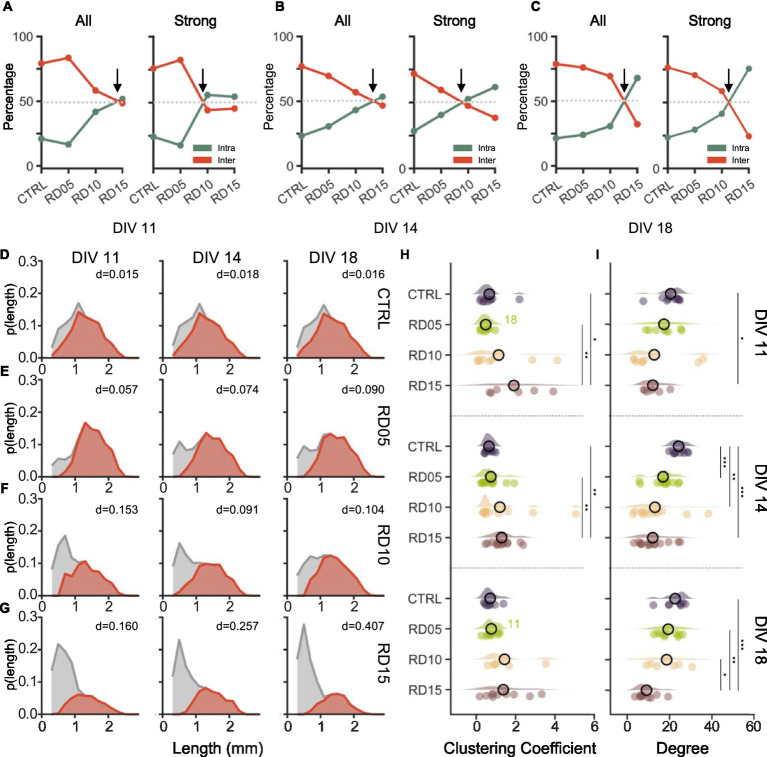
Functional topological properties are shaped by the mask removal days. Percentage of all (left) and of the strong (right) functional intra-(green) and inter-(red) compartment connections at **(A)** DIV 11, **(B)** DIV 14, and **(C)** DIV 18. We considered a connection to be strong if the absolute value of its weight was higher than a threshold set to the mean plus one standard deviation. The arrow highlights the point where a balance between the intra- and inter-compartment connections is reached (grey dotted line). **(D–G)** Probability distributions of the length of the total (grey) and of the inter- compartment (red) connections over development (from DIV 11 on the left to DIV 18 on the right) in **(D)** control, **(E)** RD05, **(F)** RD10, and **(G)** RD15 networks. The number in the panel indicated the Bhattacharyya distance between the total ant the inter-compartment length distributions at the relative time point and in the relative configuration. **(H)** Clustering coefficient and **(I)** Node Degree of the four different configurations (control, cross removal day at DIV 5, 10 and 15) over development (from DIV 11 to DIV 18). The statistical differences over development within the same configuration are indicated with the following abbreviation: 11, 14, and 18 if there was a statistical difference with DIV 11, DIV 14, and DIV 18, respectively. Refers to 0.01 < *p* < 0.05, ** to 0.001 < *p* < 0.01, and *** to *p* < 0.001 Kruskal–Wallis non-parametric test. The *p*-values are reported in Supplementary Figures S3D,E and Supplementary Figures S4D,E.

Firstly, we computed the percentage of intra- and inter-compartment connections (Figures 5A–C, left), and we then identified the strongest ones to determine which category they mainly belonged to (evaluated in terms of percentage, Figures 5A–C, right). We found that the balance between intra- and inter-compartment connections (Figures 5A–C, left dashed lines) remained consistent throughout development, falling between RD10 and RD15 at all stages. However, while RD10 assemblies tended progressively to values observed for RD05 and controls, RD15 assemblies diverged completely. Over development, intra-compartment links were always preponderant and incremented when the four sub-networks were allowed to connect. The late removal of the physical constraint strengthened the connectivity within each compartment instead of promoting inter-compartment connections, suggesting a possible lack of integration among them. It is worth noticing that we detected inconsistent inter-compartment connections at DIV 11 and DIV 14, just as in the previous paragraph, where coactivation of all four compartments was observed even before connections were allowed. However, these discrepancies could be only apparent as these results are based on the functional analysis: functional connectivity networks were derived from causality inferences mathematically obtained with cross-correlation function (cf., Materials and Methods). When an event occurs in a population, the functional link computed with the cross-correlation identifies the probability of observing another event in the other populations in a short (<50 ms) time window. Therefore, functional connections can be observed between two populations even if they are not morphologically connected.

Strong connections (Figures 5A–C, right) followed the same trend over development for all configurations except for RD10. In this case, at DIV 11 and DIV 14 (one and three days after cross removal), strong connections had a slight propensity of developing within each compartment rather than extending to others. At DIV 18, the disparity between total and strong connections was minimized, with strong connections forming mainly between compartments. This suggested that the RD10 configuration, while maintaining a good level of segregation, promoted a progressive integration of the sub-networks that reached conditions comparable to RD05 and controls only at later stages of development.

The lengths of the functional connections (shown in Figures 5D–G), both total (grey) and inter-compartment (red), supported these results. The longest connections were observed among the compartments. However, a noticeable difference was found among configurations: the long inter-compartment connections decreased as a function of the removal day of the physical constraint, as demonstrated by the lowering peak of the relative probability distribution. In the controls (Figure 5D), there was a great overlap (evaluated in terms of Bhattacharyya distance, BC) between the probability distributions of inter and total connections, which remained constant over time. RD05 networks (Figure 5E) exhibited a distribution of the inter-compartment connections similar to controls but showed disparities in the short intra-compartment ones. Additionally, the distance between the inter- and total length distributions slightly widened with maturation in favor of the short inter-compartment ones. RD10 distributions had an opposite trend (Figure 5F). In the first 2 days, there was a high probability of short total connections, which progressively decreased over time in favor of longer intra-compartment connections. As a result, the overlap between the distributions eventually resembled that of RD05 (at DIV18), with an increased number of long connections (Figure 5F, right). On the other hand, RD15 networks displayed an even more pronounced probability of short total connections, that increased over time, as demonstrated also by the increasing BC (Figure 5G).

Finally, we investigated the topological characteristics of the network by measuring the clustering coefficient (CC) and the node degree of each electrode. Throughout all stages of development, the CC (Figure 5H) appeared to increase with the removal day of the cross: RD15 assemblies had the highest CC, and controls had the lowest. At DIV 11 and DIV 14, the difference between RD15 and controls and RD05 was statistical (Supplementary Figure S4), which was expected as RD15 sub-networks were independent. The maturation of the network led to a partial rebalancing of the four configurations at DIV 18 (even though the general trend was maintained). Over time, RD05 and RD10 underwent an increase in CC and RD15 networks saw a reduction in their CC, while controls seemed unaffected. Ultimately, the mask removal at DIV 10 provides an experimental configuration where both clusterization (evaluated in terms of CC, Figure 5H) and integration (presence of long-range functional connections, Figures 5A–C), are well balanced resembling the physiological *in vivo* conditions. This topological property plays a crucial role in the optimization of the brain functions as well as in the coding processes (Sporns and Bullmore, 2014).

The degree (Figure 5I) had an opposite trend, being inversely proportional to the removal day of the physical constraint, which allowed the creation of a bigger interconnected network. In simpler terms, when the population is larger, or when more time is available to form a well-interconnected network, the degree of each node is higher. Thus, at all considered time points, controls consistently displayed the highest mean value of degree, while RD15 networks showed the lowest. These configurations differed significantly across all DIVs, with the controls standing out with the highest value at DIV 14 and RD15 being the significant lowest at DIV 18. Over time, the configurations did not show statistical differences (Supplementary Figure S3). However, multiple trends could be observed as a function of network maturation. Controls and RD05 assemblies showed some fluctuations but were stable over time. RD10 networks displayed an increase in the degree: this effect (i.e., the higher average inputs/outputs each node received/sent over time) could explain the activity observed previously, specifically the increase in network burst duration. RD15 networks showed a mild change only when interconnections occurred.

## Discussion and conclusion

In physiological conditions, segregation and integration work together harmoniously to enhance brain functions and optimize the transmission of information. According to graph theory studies, the brain’s network organization has evolved to optimize information transfer efficiency while minimizing connection costs across all temporal and spatial scales (Bullmore and Sporns, 2009). Segregation involves the specialization of different brain regions for specific tasks, ensuring efficient information processing. Integration, on the other hand, facilitates the exchange of information among the segregated regions. Functionally, this organization—typical of complex systems—was found in large-scale connectivity, giving rise to the dynamic information flow that takes place in the human brain. Additionally, it has been demonstrated that the properties of the network connectivity undergo significant fluctuations in time across different scales, inevitably affecting the balance between segregation and integration (Sporns, 2013; Fukushima et al., 2018). It has been suggested that the brain organization in terms of modularity is strongly dependent on the maturation and the aging of the networks themself, suggesting a progressive modularization in the aging brain (Meunier et al., 2009). Functional evidence of information segregation and integration is also supported by structural investigations, which have linked integrated and segregated functional states with direct structural links, especially during integration processes (Fukushima et al., 2018). Considering the maturation-dependent properties of the neuronal networks, the gradual and dynamic change of the network’s structural connections become a critical aspect to investigate. As the network learns and adapts, its connections evolve, making this slow transformation vital for its effectively functioning (Buchs et al., 1993; Bullmore and Sporns, 2009; Meunier et al., 2009). As a critical attribute of the neuronal network’s overall performance, structural change in neuronal connectivity necessitates the development of models that allow studying their effect on networks dynamics over time, for example by engineering techniques that can induce such changes at any point during cell culture (Joo et al., 2018).

### Conclusion

This goal was at the basis of the engineered *in vitro* model we presented in this work. By devising a simple and controllable physical constraint, we were able to directly control the physical connections within cortical networks and allow their formation at specific moments of network development. Our results demonstrate that structurally there exist a critical time step that allows the establishment of a well-balanced network in terms of segregation and integration of information transmission.

In general, we observed that the presence of the cross-shaped mask played a pivotal role in shaping connectivity, promoting the formation of distinct modules with varying degrees of intercommunication. In particular, the difference in removal day of this physical constraint, besides having physical implications on both the size of the overall formed network and the intrinsic connectivity among cells (Figure 2), also affected various aspects of the dynamic of the network. At different developmental stages of the culture, firing and bursting parameters showed variations and followed the same trend (without any statistical difference, Supplementary Figure S6) as a function of the temporal distance from the day of the physical constraint removal. This suggested that the level of maturation of the network and its rhythm over culture time was highly influenced by how much time the different modules had to interact. The rates and the percentages of random spiking (Figures 3A,B,D) were altered when the previously established equilibrium was disrupted by the creation of new connections among the different compartments. This was not the case of the mean frequency intra burst (Figure 3C), which suggests that the “density” of the bursts in terms of number of spikes is controlled by other mechanisms beyond the formation of new communication pathways among assemblies.

Already from the trends and interplay among these parameters, we were able to identify a time point in the development of the network that seemed to establish a balance between integration, in this case evaluated in terms of rates of activity, and segregation of information, as proved by the scattering of random spiking data. It is the case of the physical constraint removal at DIV 10. Only at this developmental stage, the removal of the cross-shaped mask allowed for an interesting balance among segregation and integration, already from a morphological point of view (Figure 2). Despite RD10 assemblies started as highly segregated sub-networks (Figures 4D–5A), this configuration favored the regeneration over time of a strong connectivity (Figure 5C, right) among a high number of compartments with long links (Figure 5F, right): at DIV 11 each network bursts mainly involved one or two compartments, but at DIV 18 the peak shifted to 4C (Figure 4D), thereby facilitating the integration of information. Finally, another consequence of the timing of the physical constraint removal concurrently with network formation was a higher instability within the overall network, resulting in an upward trend in network burst duration, accompanied by an increased variability.

If the physical constraint is removed before this stage, as happens for RD05 cultures, cells are in a highly premature stage of their development which favored the growth of new connections. The resulting overall network is very similar to controls, both morphologically (Figure 2A) and functionally across all DIVs (Figure 5). The high connectivity among compartments (Figures 5A–C) promoted the involvement of the entire network during the population events already from early days of development (DIV 11), just as in control conditions. This evidence suggested that when the physical constraint was removed at initial stage of development the networks were able to functionally recover their ability to connect and integrate information as if no obstacles took place.

Conversely, the late removal of the physical constraint (in our experimental model represented by RD15 networks) hindered on some level the creation of a strong connectivity among the four compartments, whose trend of maturation seemed to be unaffected by the creation of the inter-population connections. Indeed, RD15 was the only configuration that could not create a uniform network, which remained more clustered and with more segregated assemblies (Figure 2C). Functional analysis revealed that the probability of forming connections among compartments was the lowest (Figures 5A–C,G; Supplementary Figure S7) and the clustering coefficient was the highest (Figure 5H), thus indicating a strong tendency of the sub-networks to exhibit an uncorrelated dynamic (Figure 4E).

In conclusion, our model allowed us to state that a critical time frame exists in culture development for maintaining a correct expression of both segregation and integration, morphologically and functionally. This result is in line with previous literature where smaller clusters of neurons were cultured on a micropatterned surface and allowed to communicate at different time steps: assemblies were able to form an interconnected network up to DIV 10 (Joo et al., 2018).

### Limitation of the model

Our experimental set-up allowed us to isolate and examine key mechanisms underlying the development of neuronal networks, with a focus on the functional and structural properties of segregation and integration. However, due to its intrinsic simplicity, such an *in vitro* model is subject to certain limitations. Firstly, the presented model does not include guidance for neurite outgrowth, resulting in limited control over spatial distribution. Nevertheless, this limitation provided the opportunity to investigate the self-assembly properties in the topological organization of networks, both structurally and functionally. Our model facilitates the establishment of a compartmentalized network featuring different segregation and integration levels. The adjustment of these levels is achievable through modifications to the timing of the PDMS mask removal. However, it is important to note that this method does not account for the ability to selectively treat an individual compartments with specific compounds. Achieving this capability would necessitate the implementation of a microfluidic system specifically designed to separate the medium. These systems facilitate specific connections between different compartments through microstructures (microchannels) allowing for the thorough examination of the level of segregation and integration imposed by the structure itself. Indeed, varying the number of microchannels provides the opportunity to observe the changes in interaction between compartments modulating the segregation/integration levels of the networks. However, it is important to note that this approach does not enable the study of the network’s capacity to self-organize. In other words, it is impossible to restrict the connection between compartments during the early stages of development, thereby preventing the observation of how the network might naturally evolve without imposed restrictions.

Also, the achieved results came from planar interconnected networks. Nowadays it is recognized how three-dimensionality plays a fundamental role in shaping the electrophysiological patterns of activity (Dingle et al., 2020; Callegari et al., 2024). Moreover, our model does not take into account the cell heterogeneity presents in the brain, as it does not yet reproduce the circuits (such as the cortico-hippocampal or the cortico-striatal-thalamic ones) involved in many higher brain functions. However, the intrinsic simplicity of the proposed method guarantees adaptability, versatility, and suitability for more complex models that mimic these more realistic *in vivo* features.

### Exploitation and perspectives

Despite (and perhaps precisely because) of our model simplicity, our *in vitro* model holds significant promise in increasing our comprehension of the complex mechanisms involved in segregation and integration during brain development. By providing a controlled and reproducible environment, it enables researchers to investigate the cellular and molecular interactions involved in these processes and identify the potential anomalies that lead to defects in nervous cell segregation and integration. As such, our *in vitro* model could have significant implications for neurological developmental disorders, such as autism and schizophrenia, providing insights into dysfunction effects related to cell migration, neuronal differentiation, and brain circuit formation. An imbalance between segregation and integration can also arise in an advanced stage of brain development, potentially contributing to late-onset conditions such as cancer. Indeed, cancer can disrupt the physiological levels of brain segregation and integration both directly through tumor invasion into the brain and indirectly via systemic effects. Tumor infiltration into surrounding brain tissues interrupts normal cell segregation patterns, resulting in nervous circuit disorganization and integration loss. Additionally, cancer’s systemic nature can trigger inflammatory responses and molecule production (e.g., cytokines), further altering brain segregation and integration patterns. Finally, integration and segregation may also be affected after localized abnormalities or injuries, often related to disruptive effects, such as focal epilepsy or stroke. Seizures or ischemic events in localized brain regions may destroy the normal connectivity and communication between neurons in that area (Cerutti and Brofiga, 2024). This disruption has been proven to interfere with the specialized processing that occurs within interconnected brain regions, leading to a loss of integration and an increased segregation (Pedersen et al., 2015; Guo et al., 2019). Our model could be a valid experimental platform to investigate all these aspects and to test how possible therapeutic approaches act on restoring this important balance.

## Data Availability

The datasets presented in this study can be found in online repositories. The names of the repository/repositories and accession number(s) can be found below: https://zenodo.org/doi/10.5281/zenodo.10998780.
