# Supplementary material for: Investigating the interplay between segregation and integration in developing cortical assemblies
Source: Front Cell Neurosci. 2024 Sep 12;18:1429329. doi: 10.3389/fncel.2024.1429329 (PMC11424435; doi:10.3389/fncel.2024.1429329)
Supplement: Supplementary file 1 [file Data_Sheet_1.pdf]

Investigating the interplay between segregation and integration in developing cortical assemblies

Valerio Barabino[1†], Ilaria Donati della Lunga[1†], Francesca Callegari[1], Letizia Cerutti[1,2], Fabio Poggio[1], Mariateresa Tedesco[1], Paolo Massobrio[1,3], Martina Brofiga[1,2,4, *]

[1] Department of Informatics, Bioengineering, Robotics and Systems Engineering (DIBRIS), University of Genova, Genova, Italy

[2] Neurofacility, Istituto Italiano di Tecnologia, Genova, Italy

[3] National Institute for Nuclear Physics (INFN), Genova, Italy

[4] ScreenNeuroPharm s.r.l, Sanremo, Italy

[†] Contributed equally.

*** Correspondence:**
Martina Brofiga
martina.brofiga@unige.it

Keywords: cortical networks, segregation, integration, Micro-Electrode Arrays, PDMS device, connectivity.

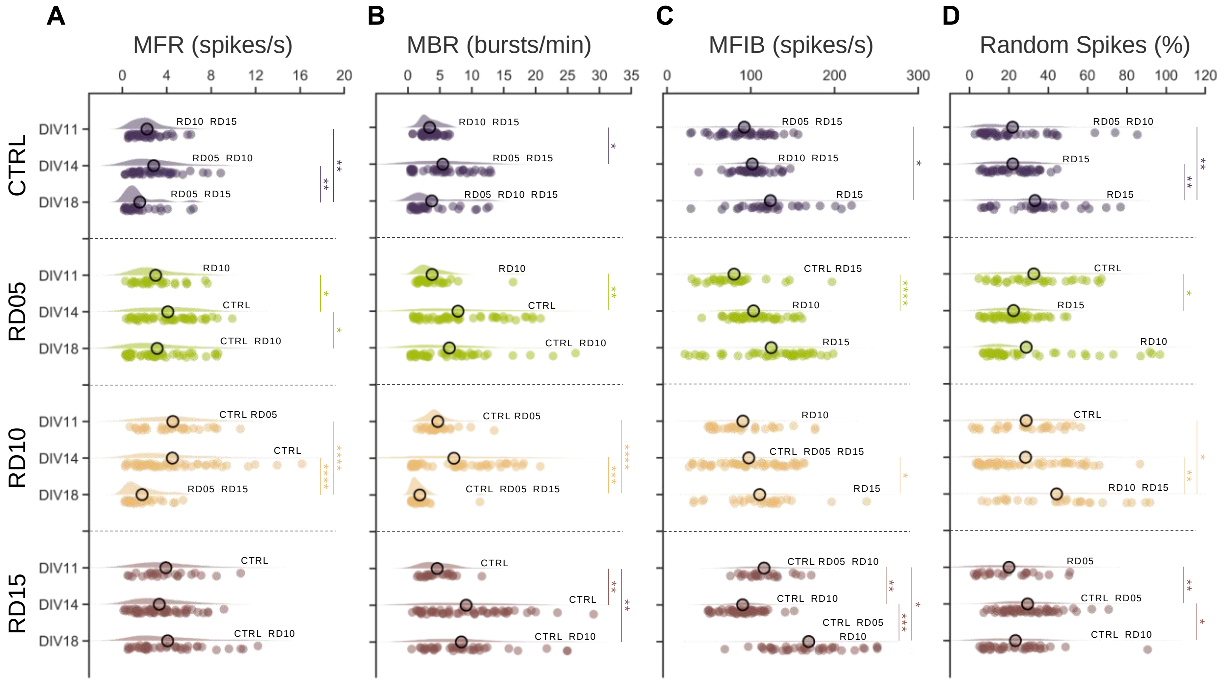

Figure S1: Spiking and bursting parameters. (A) Mean firing rate (MFR), (B) mean bursting rate (MBR), (C) mean frequency intra burst (MFIB), and (D) percentage of random spiking of the four different configurations (control, cross removal day at DIV 5, 10 and 15) over development (from DIV 11 to DIV 18). The statistical differences between different configuration at the same DIV are indicated with the following abbreviation: CTRL, RD05, RD10 and RD15 if there was a statistical difference with CTRL, RD05, RD10 and RD15, respectively. * refers to $0.01 < p < 0.05$, ** to $0.001 < p < 0.01$, *** to $0.0001 < p < 0.001$, and **** to $p < 0.0001$ Kruskal-Wallis non-parametric test.

A

MFR (spikes/s)	DIV11	DIV14	DIV18
CTRL	2.2 ± 1.3	2.8 ± 2.0	1.6 ± 1.6
RD05	3.0 ± 1.9	4.1 ± 2.4	3.1 ± 2.5
RD10	4.6 ± 2.4	4.5 ± 3.6	1.8 ± 1.4
RD15	3.9 ± 2.5	3.3 ± 2.1	4.1 ± 3.0

B

MBR (bursts/min)	DIV11	DIV14	DIV18
CTRL	3.4 ± 1.4	5.5 ± 3.9	3.7 ± 3.7
RD05	3.8 ± 2.8	7.8 ± 5.8	6.5 ± 5.9
RD10	4.7 ± 2.3	7.2 ± 6.1	1.9 ± 2.0
RD15	4.6 ± 2.4	9.1 ± 6.3	8.4 ± 5.9

C

MFIB (spikes/s)	DIV11	DIV14	DIV18
CTRL	92.0 ± 29.8	101.6 ± 20.5	123.3 ± 40.9
RD05	79.9 ± 33.5	103.2 ± 26.5	124.3 ± 46.4
RD10	90.5 ± 35.2	97.5 ± 40.4	110.5 ± 43.9
RD15	115.7 ± 24.3	90.1 ± 21.7	169.0 ± 48.1

D

RS (%)	DIV11	DIV14	DIV18
CTRL	21.9 ± 18.7	22.0 ± 10.3	33.3 ± 18.0
RD05	32.7 ± 18.1	22.4 ± 10.4	28.8 ± 25.4
RD10	28.8 ± 15.8	28.5 ± 21.2	44.3 ± 24.4
RD15	20.1 ± 14.5	29.5 ± 13.2	23.3 ± 15.8

E

NBD (ms)	DIV11	DIV14	DIV18
CTRL	937.2 ± 136.9	820.4 ± 101.3	715.2 ± 100.8
RD05	805.7 ± 85.2	749.6 ± 79.9	636.1 ± 106.1
RD10	714.1 ± 54.5	751.2 ± 142.3	833.8 ± 136.4
RD15	750.5 ± 143.7	718.9 ± 100.9	505.9 ± 88.9

Figure S2: Mean and Standard deviation of different parameters. A) mean firing rate (MFR), (B) mean bursting rate (MBR), (C) mean frequency intra burst (MFIB), (D) percentage of random spiking (RS) and (E) network burst duration (NBD), within each configuration (control, RD05, RD10, RD15) over development (from DIV 11 to DIV 18).

A (rows: MFR; columns: MBR)

CTRL	DIV11	DIV14	DIV18
DIV11		0.016615	0.149488
DIV14	0.259904		0.096459
DIV18	0.004809	0.001285	

RD05	DIV11	DIV14	DIV18
DIV11		0.0033	0.097758
DIV14	0.047664		0.207673
DIV18	0.882884	0.033015	

RD10	DIV11	DIV14	DIV18
DIV11		0.422925	2.32E-08
DIV14	0.433668		0.000627
DIV18	5.39E-06	7.36E-05	

RD15	DIV11	DIV14	DIV18
DIV11		0.003285	0.003433
DIV14	0.377822		0.597748
DIV18	0.802885	0.435205	

B (rows: RS; columns: MFIB)

CTRL	DIV11	DIV14	DIV18
DIV11		0.086951	0.031896
DIV14	0.19873		0.190628
DIV18	0.004357	0.003447	

RD05	DIV11	DIV14	DIV18
DIV11		7E-05	0.359102
DIV14	0.012884		0.312931
DIV18	0.082373	0.63848	

RD10	DIV11	DIV14	DIV18
DIV11		0.696223	0.126469
DIV14	0.726327		0.04938
DIV18	0.012275	0.005538	

RD15	DIV11	DIV14	DIV18
DIV11		0.004661	0.030849
DIV14	0.007194		0.000413
DIV18	0.291915	0.011759	

C (rows: NBD; columns: NBD)

CTRL	DIV11	DIV14	DIV18
DIV11		0.067121	0.001243
DIV14	ns		0.050044
DIV18	**	ns	

RD05	DIV11	DIV14	DIV18
DIV11		0.147501	0.004993
DIV14	ns		0.007059
DIV18	**	**	

RD10	DIV11	DIV14	DIV18
DIV11		0.784846	0.105193
DIV14	ns		0.15634
DIV18	ns	ns	

RD15	DIV11	DIV14	DIV18
DIV11		0.451566	0.00574
DIV14	ns		0.000293
DIV18	**	***	

D (rows: STRONG; columns: ALL)

CTRL	DIV11	DIV14	DIV18
DIV11		0.2505	0.41175
DIV14	0.45016		0.62237
DIV18	0.57674	0.2786	

RD05	DIV11	DIV14	DIV18
DIV11		0.0406	0.00592
DIV14	0.0169		0.92268
DIV18	0.0647	0.62749	

RD10	DIV11	DIV14	DIV18
DIV11		0.78845	0.14891
DIV14	0.69807		0.40139
DIV18	0.19338	0.33292	

RD15	DIV11	DIV14	DIV18
DIV11		0.87153	0.22699
DIV14	0.60796		0.15649
DIV18	0.17623	0.1429	

E (rows: CC; columns: DEGREE)

CTRL	DIV11	DIV14	DIV18
DIV11		0.094041	0.098648
DIV14	0.341025		0.56326
DIV18	0.408961	1	

RD05	DIV11	DIV14	DIV18
DIV11		0.828004	0.656853
DIV14	0.218266		0.535143
DIV18	0.032969	0.576738	

RD10	DIV11	DIV14	DIV18
DIV11		0.396066	0.121335
DIV14	0.354539		0.111341
DIV18	0.15558	0.133994	

RD15	DIV11	DIV14	DIV18
DIV11		0.804571	0.103742
DIV14	0.620691		0.319533
DIV18	0.515181	0.725345	

Figure S3: P-values over development. P-values tables from Kruskal-Wallis non-parametric test: (A) mean firing rate (MFR, left) and mean bursting rate (MBR, right), (B) percentage of random spiking (RS, left) and mean frequency intra burst (MFIB, right), (C) network burst duration (NBD), (D) strong (left) and all (right) connections, (E) clustering coefficient (CC, left) and total degree (right) within each configuration (CTRL, RD05, RD10, RD15) over development (from DIV 11 to DIV 18). * refers to $0.01 < p < 0.05$, ** to $0.001 < p < 0.01$, and *** to $p < 0.001$.

A

MFR / MBR

DIV11	CTRL	RD05	RD10	RD15
CTRL		0.704876	0.008273	0.048118
RD05	0.094369		0.018118	0.075097
RD10	3.4E-05	0.012043		0.753079
RD15	0.002354	0.136198	0.289315	

DIV14	CTRL	RD05	RD10	RD15
CTRL		0.043928	0.143389	0.003026
RD05	0.007276		0.450767	0.257905
RD10	0.016195	0.948165		0.083477
RD15	0.211329	0.099384	0.170327	

DIV18	CTRL	RD05	RD10	RD15
CTRL		0.038232	0.024307	4.97E-05
RD05	0.004973		9.36E-05	0.05245
RD10	0.533725	0.02308		4.72E-09
RD15	1.5E-05	0.199793	0.000421	

B

RS / MFIB

DIV11	CTRL	RD05	RD10	RD15
CTRL		0.012909	0.198924	0.019116
RD05	0.004357		0.445777	5.05E-06
RD10	0.043386	0.591208		0.000453
RD15	0.847352	0.008476	0.05273	

DIV14	CTRL	RD05	RD10	RD15
CTRL		0.227475	0.000758	0.043167
RD05	0.953091		0.009901	0.375272
RD10	0.480245	0.61209		0.032892
RD15	0.005963	0.005148	0.237783	

DIV18	CTRL	RD05	RD10	RD15
CTRL		0.103174	0.536479	0.031106
RD05	0.106272		0.389103	0.006787
RD10	0.072988	0.004178		0.024559
RD15	0.00676	0.775095	0.000288	

C

NBD / NBD

DIV11	CTRL	RD05	RD10	RD15
CTRL		0.038989	0.000524	0.041575
RD05	*		0.035692	0.487453
RD10	***	*		0.417887
RD15	*	ns	ns	

DIV14	CTRL	RD05	RD10	RD15
CTRL		0.192792	0.127908	0.0407
RD05	ns		0.662303	0.369486
RD10	ns	ns		0.770931
RD15	*	ns	ns	

DIV18	CTRL	RD05	RD10	RD15
CTRL		0.087375	0.124822	0.00095
RD05	ns		0.007546	0.005258
RD10	ns	**		0.000489
RD15	***	**	***	

D

STRONG / ALL

DIV11	CTRL	RD05	RD10	RD15
CTRL		0.1372	0.00095	0.00876
RD05	0.1372		0.00076	0.00705
RD10	0.00433	0.01068		0.6911
RD15	0.08738	0.03844	0.96473	

DIV14	CTRL	RD05	RD10	RD15
CTRL		0.82667	0.00468	0.0015
RD05	0.32442		0.04469	0.00988
RD10	0.00549	0.17607		0.35487
RD15	0.0038	0.0224	0.32596	

DIV18	CTRL	RD05	RD10	RD15
CTRL		0.54297	0.01324	0.00006
RD05	0.22363		0.07022	0.00011
RD10	0.01664	0.24657		0.00693
RD15	0.00019	0.00012	0.00428	

E

CC / DEGREE

DIV11	CTRL	RD05	RD10	RD15
CTRL		0.363722	0.082915	0.0208
RD05	0.508883		0.092892	0.121335
RD10	0.741182	0.916359		0.196706
RD15	0.011985	0.004509	0.121335	

DIV14	CTRL	RD05	RD10	RD15
CTRL		0.000864	0.004639	0.000459
RD05	0.884836		0.064406	0.065184
RD10	0.805541	0.78565		0.918067
RD15	0.001238	0.007609	0.071828	

DIV18	CTRL	RD05	RD10	RD15
CTRL		0.091374	0.245278	0.000734
RD05	0.593955		0.587594	0.001152
RD10	0.052808	0.158526		0.012606
RD15	0.091374	0.150927	0.664389	

Figure S4: P-values between different configurations. P-values tables from Kruskal-Wallis non-parametric test: (A) mean firing rate (MFR, left) and mean bursting rate (MBR, right), (B) percentage of random spiking (RS, left) and mean frequency intra burst (MFIB, right), (C) network burst duration (NBD), (D) strong (left) and all (right), (E) clustering coefficient (CC, left) and total degree (right) between the four different configurations (CTRL, RD05, RD10, RD15) at each DIV (11, 15, 18). * refers to $0.01 < p < 0.05$, ** to $0.001 < p < 0.01$, and *** to $p < 0.001$.

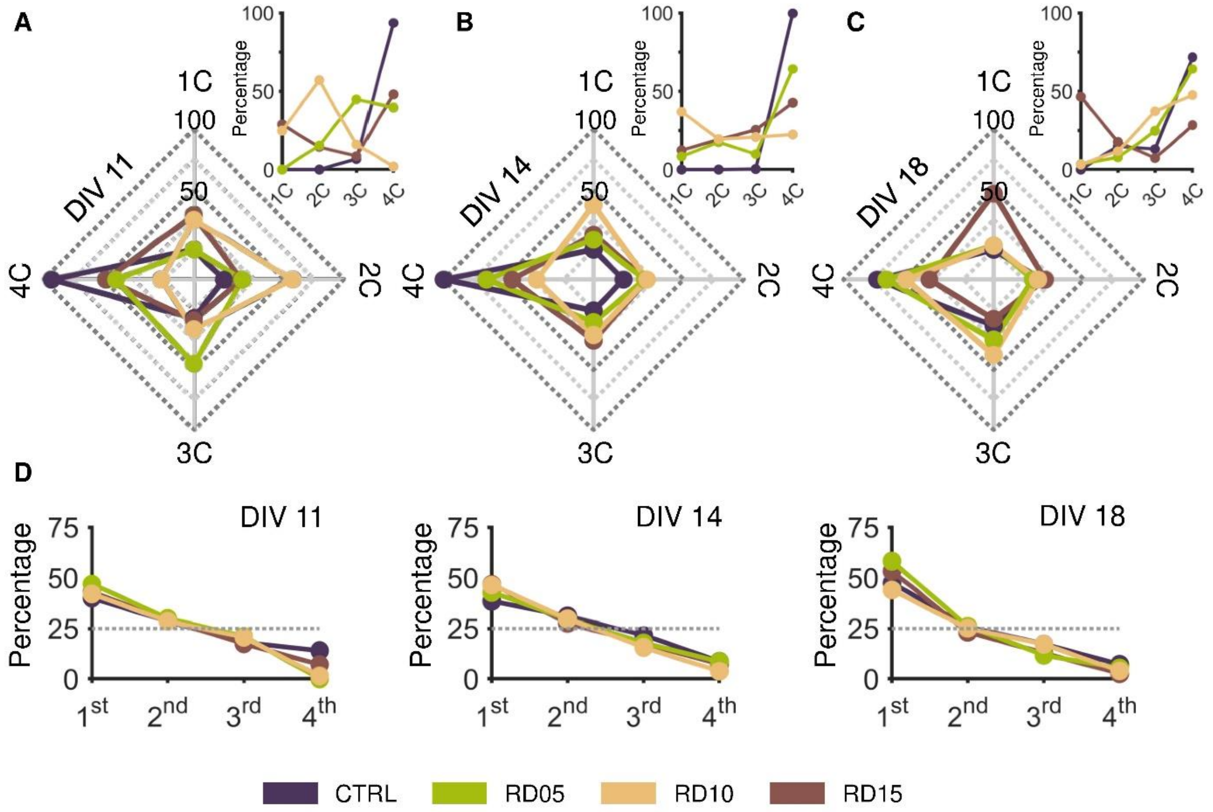

Figure S5: Network burst activity propagation as a function of development. (A-C) Radar plots showing the percentage of times network bursts involve from one single (1C) to all four (4C) compartments in different configurations (controls, RD05, RD10 and RD15) at (A) DIV 11, (B) DIV 14 and (C) DIV 18. The insets offer a different visualization of the same quantity for the sake of clarity. (D) Percentage of times a compartment is the initiator of a network burst event between configurations (CTRL, RD05, RD10 and RD15) at (A) DIV 11, (B) DIV 14 and (C) DIV 18. We counted the times that the leader (initiating electrode) of a network event fell within the different compartments and consequently ordered them in a hierarchical way so that the compartment that initiated the most network events was labelled as 1st, up to the last which was labelled as 4th. The dotted grey line indicates the random condition where there is not a leader compartment, i.e., all compartments have the same probability of giving rise to a network burst. The different configuration conditions are color-coded as in the legend.

A

DIV 18 (columns); DIV 14 (rows)

MFR	RD05	RD15
CTRL	0.85119	
RD10		0.64214

B

DIV 18 (columns); DIV 14 (rows)

MBR	RD05	RD15
CTRL	0.6253	
RD10		0.22421

Figure S6: P-values over development in different configurations. P-values tables from Kruskal-Wallis non-parametric test: comparison between DIV 14 and 18 of (A) mean firing rate (MFR) and (B) mean bursting rate (MBR) between two couple of configurations (CTRL - RD05 and RD10 - RD15).

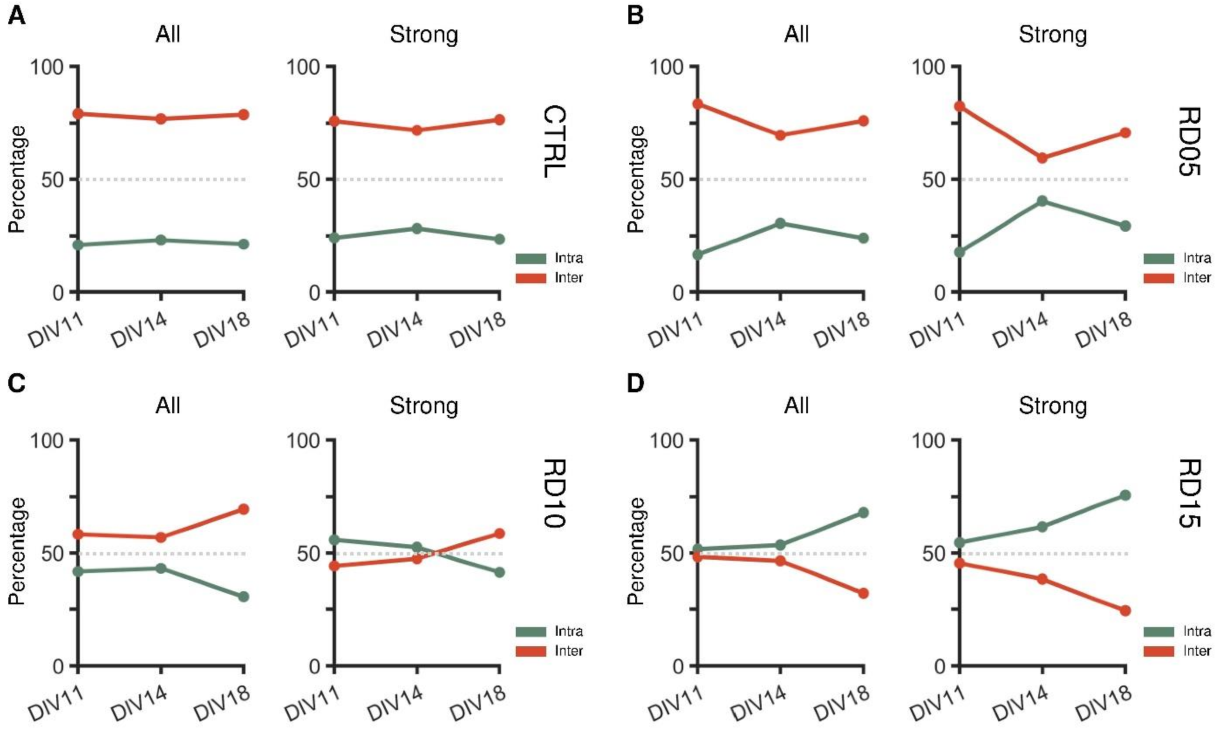

Figure S7: Functional connectivity as a function of development. Percentage of all (left) and of the strong (right) functional intra- (green) and inter- (red) compartment connections over development (from DIV 11 to DIV 18) in (A) controls (CTRL), (B) RD05, (C) RD10, and (D) RD15 configurations. We considered a connection to be strong if the absolute value of its weight was higher than a threshold set to the mean plus one standard deviation. The grey dotted line figure out the balance between the intra- and inter- compartment connections.
